# The relationship between nutrition reading and label use and nutrition knowledge amongst a sample of rural youth studying at a university in South Africa

**DOI:** 10.4102/hsag.v24i0.1320

**Published:** 2019-11-12

**Authors:** Nomasonto Xazela, Willie T. Chinyamurindi, Herring Shava

**Affiliations:** 1 Department of Livestock and Pasture Science, University of Fort Hare, Alice, South Africa; 2 Department of Business Management, University of Fort Hare, Alice, South Africa

**Keywords:** rural, youth, consumer nutrition label use, nutrition knowledge, nutrition reading

## Abstract

**Background:**

Within rural communities, quests for understanding consumer behaviour patterns become key, especially given the challenges that exist in such communities. Furthermore, youth consumers are an important cohort in rural communities in South Africa. Yet little empirical work has been conducted in studying their consumptive habits (usually in favour of youths in urban areas).

**Aim:**

This study sought to measure the relationship between nutrition label use and nutrition knowledge amongst a sample of rural youth. This also included ascertaining the roles of demographic variables such as (1) gender and (2) and the number of years in tertiary education in this relationship.

**Setting:**

The study was located within a South African rural context using a sample of rural youths.

**Method:**

A quantitative study was utilised by means of a self-administered questionnaire, involving 150 youths residing in a rural community located in the Eastern Cape province of South Africa. Inferential statistical analysis was conducted using correlation tests, analysis of variance and linear regression.

**Results:**

Three main findings emerged. Firstly, rural youth consumers’ nutrition label use had no significant relationship with nutrition knowledge. Secondly, no significant difference existed by gender concerning nutrition knowledge. Finally, the level of education amongst the rural youth consumers had no relationship with aspects of nutrition knowledge.

**Conclusion:**

Based on the findings, recommendations are made not only for theory and practice but also for policy in relation to both the rural context and for optimal health.

## Introduction

Nutrition knowledge and understanding the importance thereof is regarded as a major means of encouraging consumers in making healthier choices (Deshmukh & Goyal 2017). Key to this understanding is the interaction that exists between individuals and their environment (Grunert et al. 2010). Quests in understanding how to improve nutrition knowledge acquisition can be a useful basis not only for community interventions to challenges such as obesity, but also to ensure a sustainable livelihood (Flynn 2015). In the extant literature, calls exist for more research exploring how aspects of individual and societal behaviour relate to nutrition knowledge (Kabir, Miah & Islam 2018).

Furthermore, the role of nutrition label use in nutrition knowledge, despite being an under-explored focus area in rural communities, can be a worthwhile investigation based on three reasons. Firstly, rural communities are overburdened with challenges such as poverty (Van Schalkwyk 2015). Thus, poverty exists as a potential deterrent to consumptive habits. This presents a need to seek ways of curtailing such limitations. Secondly, because of the aspect of poverty, rural inhabitants are limited in terms of food consumption. This is compounded by a lack of knowledge concerning the difference between healthy and unhealthy food (Hendriks & McIntyre 2016). Finally, a general observation in South African rural communities is the role that biographical factors can play, not only in the acquisition of knowledge concerning nutrition but also regarding responses to nutrition interventions (Kimani-Murage et al. 2010).

### Problem statement

Young people in South Africa currently face a myriad of challenges, predominantly unemployment (Coetzee 2014). Furthermore, South Africa, like the rest of the African continent, is argued to have a young yet also growing population (Page 2012). One key risk that youths face has to do with aspects of the choices made concerning their health (Amy 2019). A call exists (World Health Organization 2014) to prioritise understanding around the challenges concerning aspects of health of the youth. The ramification here is that youths are the future leaders of economies and hence the focus to help them charter and occupy this position through better healthcare.

Issues that could be of interest here are to understand the networks youths use to access information about health (Hampshire et al. 2015). Narrowly, the interest here is on understanding issues concerning nutrition knowledge and how it relates to the youth market. Therefore, the purpose of this study was to identify and prioritise issues about nutrition label use, nutrition knowledge and, based on the findings, to propose ways in which rural inhabitants can live not only sustainably but also with a development focus in mind. This can be a useful precursor to interventions that find solutions to nutrition-related challenges evident in rural communities.

## Literature review

### Nutrition knowledge

Nutrition knowledge has been reported to mediate the relationship between socio-economic status and diet quality (Psaltopoulou et al. 2017). Regular and adequate supply of a variety of foods can be ensured through financial security, the lack of which leads to lower levels of malnutrition (under- and over-nutrition). In addition, the risk of developing chronic diseases because of poor lifestyle will be mitigated (Pretorius & Sliwa 2011).

The availability of and access to nutritious, diverse and balanced diets were identified as key constraints in achieving food and nutrition security and overall to human health and well-being (Govender et al. 2017). Calls have been made for a more expansive understanding of issues of nutrition knowledge and how this relates to aspects such as decision-making and human behaviour (Miller & Cassady 2015).

### Influence of demographics on nutrition knowledge

Food cost is noted as a contributor towards socio-economic patterning and choices of a healthy diet. Higher occupational social class is significantly associated with greater food expenditure, which in turn is associated with healthier purchasing (Pechey & Monsivais 2016). With respect to gender and education levels, it has been established that generally women and people with higher education levels have good nutrition knowledge (Laz et al. 2015).

Concerning age, a positive link appears to exist between the level of nutrition knowledge (Romanos-Nanclares et al. 2018) and age, with adult consumers having a better understanding of nutrition knowledge than young people. Furthermore, studies have shown that maternal access to nutrition is important during childhood years and later age (Jones et al. 2014). The thinking here is that access to good nutritious food in childhood sets the individual for a healthy life, both during adolescence (Govender et al. 2018) and later in adult life (Grosso et al. 2012). Based on all the presented literature, the following hypotheses were set:

Hypothesis 1: Women have higher nutrition knowledge scores compared to men.

Hypothesis 2: The number of years of tertiary education has a significant effect on the nutrition knowledge of the youth.

### Nutrition label use and nutrition knowledge

As consumers have become gradually selective about what they consume and how it affects their health, the food industry has responded by providing more detailed nutrition information on food labels (Petrovici et al. 2012). In 2010, South Africa and other African countries took bold steps to enact a law for publishing food contents on labels (Kempen et al. 2012). Despite these legislative efforts, consumers continue to struggle in making decisions concerning food choices (Miller et al. 2017). These challenges may be the cause of individual knowledge processing and not necessarily of the use of nutrition labels. Therefore, it is hypothesised that:

Hypothesis 3: Nutrition label use is positively related to nutrition knowledge.

### Reading of nutrition information and nutrition knowledge

Reading the nutrition information may assist an individual in making healthier food choices (Azman & Sahak 2014). Some consumers experience challenges when reading nutrition labels, such as the font size of nutrition information being too small and understanding terminology used in the ingredients list (Koen et al. 2016). However, Van der Merwe et al. (2013) reported that South African consumers selectively read nutrition information on food labels. Some consumers added that they were unsure of their understanding of the information provided (Kempen et al. 2012). Not reading the nutrition label may be because some consumers do not understand all the information or they are unwilling to read or to invest time in understanding it (Guthrie, Mancino & Lin 2015). The challenges may be in understanding the numerical information and differences in amount of fats or other nutrients when comparing products (Miller et al. 2015). Thus, the lack of reading of nutrition labels may be attributed to the lack of exposure and understanding (Matthews, Doerr & Dworatzek 2015). The primary goal should be to provide clear guidance on how best to select food items for a healthy diet (Moore et al. 2018). Hence, this study hypothesised that:

Hypothesis 4: Reading of nutrition labels is significantly related to nutrition knowledge.

## Methodology

The study followed a quantitative methodology utilising a self-administered questionnaire. The motivation behind quantitative research is to produce information and enable an understanding about the social world (Antwi & Hamza 2015). It gives answers to questions regarding the recurrence of a phenomenon, or the extent to which the phenomenon affects the sample population. The advantage of a self-administered questionnaire is that it allows users to collect a vast amount of data in a short period of time and economically (Picincu 2018).

### Sample and procedure

Data were gathered through a self-administered questionnaire targeted at youth respondents residing in a rural area of the Eastern Cape province of South Africa. The justification for this is that a rural community under study faces significant challenges that relate to aspects of health and where nutrition plays an important role (Parliamentary Monitoring Group 2016). A non-probability convenience sampling approach was utilised because of the difficulty of accessing a reliable sample base. Furthermore, the researchers were impeded by challenges such as costs of conducting the research and hence the use of such a sampling approach and technique. The survey was conducted in September 2018, where the selection of participants was done randomly. The participants were approached and asked if they are interested about the study. When the consumer agreed to be part of the study, the enumerator explained the purpose of the study and the interviewer signed the consent form. These respondents were university students who were studying at a historically black institution, which is based in a rural area. The final number of respondents who participated in the study was 150, of which 65 were men and 85 were women.

### Instrument

The survey instrument was composed of three sections. The first section consisted of demographic questions related to the respondents’ gender, years of study at a tertiary institution, nationality and age. The second section of the questionnaire had questions related to food nutrition label use adapted from an international study (De Magistris, Gracia & Barreiro-Hurle 2010). The items as guided by De Magistris et al. (2010) were split into three groups: (1) ingredients use, (2) nutrition facts and (3) nutrition claims. In testing the reliability, the nutrition label use scale had a Cronbach’s alpha coefficient of 0.73, which is above the recommended threshold of 0.7 (Nunnally 1978). An example item of the nutrition label use scale was the following: ‘as a consumer, I use nutrition labels when shopping’ and this was measured using a four-point Likert scale, where 1 = never; 2 = once in a while; 3 = often; and 4 = always.

The final section of the questionnaire had questions concerning nutrition knowledge adapted from an international study (Krause et al. 2018) consisting of 12 items measured on a four-point Likert scale (1 = disagree strongly to 4 = agree strongly). An example item is as follows: ‘when I have questions on healthy nutrition, I know where I can find information on this issue’. In testing the reliability, the nutrition label use scale had a Cronbach’s alpha coefficient of 0.73, which is above the recommended threshold of 0.7 (Nunnally 1978). Barreirro-Hurle et al. (2010) validated these measures mentioned above through pilot surveys.

### Data analysis

The Statistical Package for Social Sciences (SPSS) 2018 version 24 was used for data analysis (IBM 2016). Descriptive statistics were tested especially with the biographical data. Inferential statistics were tested through the independent samples *t*-test, the analysis of variance (ANOVA) and multiple linear regression. In other words, the study combined hypotheses 3 and 4 to determine if they predict nutrition knowledge. Initially, the hypotheses were formulated independently during the literature review with the goal of bringing to light the ongoing discussion about these variables.

### Ethical consideration

Ethical approval for the study was obtained from the University of Fort Hare Research Ethics Committee (UREC) (ethical clearance number: CHI021).

## Results

The results of the study are presented and discussed in two sections. The first section focuses on the study’s descriptive data. Table 1 provides a detailed description and means of variables used in the analysis of data. The data are presented with means for the two independent variables: nutrition labels and the nutrition knowledge. Years of study were excluded from the table although their combined mean was observed as 3.11.

**TABLE 1:** Description and means of the variables.

Variables	Description	Mean
**Independent variables**		
Nutrition labels†	Are you aware of food labels? (NLU1)	1.39
	Do you read the nutrition information on the labels before placing food products into your shopping basket prior to purchasing? (NLU2)	1.33
	How often do you choose products with nutrition labels over products without such labels? (NLU4)	2.53
	When shopping, how important is the nutrition information about food ingredients for you? (NLU5)	3.51
	When shopping, how important is standardised labelling on food packages for you? (NLU6)	3.33
	When shopping, how important are production and expiration dates for you? (NLU7)	3.91
	Nutrition information is the major factor that determines the choice of food. (NLU8)	4.48
	Reading of production and expiration date on food package is important for health. (NLU9)	4.83
	Unhealthy food packaging without standard sign and health license not to be used. (NLU10)	4.61
Reading of nutrition information (self-confidence)‡	I can easily understand the nutrition facts (e.g. amount of energy, sugar, protein, etc.) on food packages (know_a1)	1.73
	I can easily understand nutrition issues I read about in newspapers and brochures (know_a2)	1.54
	I can understand nutritionist recommendations about health and nutrition requirements that are appropriate for my age group (know_a3)	1.78
	Boiling is one of the more healthy cooking methods (know_a4)	1.87
	I can understand information and recommendations about proper nutrition for children in the media (e.g. TV, Internet, radio, etc.) (know_a5)	1.61
	Daily physical activity for 30 ± 40 min prevents obesity (know_a6)	1.65
	I know how different vegetables are cultivated and grown (know_a7)	1.57
	Daily eating breakfast helps me to learn more (know_a8)	1.51
	Unhealthy food packing without standard sign and health license should not be used (know_a9)	1.76
	Consumption of salty snacks (e.g. chips, corn puffs, etc.) is harmful for health (know_a10)	1.73
	Excessive consumption of sugar, sweets and chocolate is harmful for health (know_a11).	1.94
	I have enough power to resist unhealthy foods (e.g. fast food, pizza, carbonated drinks, etc.) (KK1).	2.1
	If I go to restaurant or fast-food outlets with my friends, and all of them choose unhealthy foods (e.g. pizza, French fries, carbonated drinks, etc.), I am able to choose healthy foods (KK2).	1.87
	I can easily say ‘no’ to unhealthy eating suggestions from my friends (KK3).	1.79
	If I encounter unhealthy behaviour at home, school or in other settings, I am able to challenge them (KK4).	1.97
	If my parents or family prepares unhealthy snacks (e.g. chips, fruit roll-ups, corn snacks etc.) for me to take to school, I accept them (KK5).	2.14
	If my family were overweight and eating a high-fat diet, I would tell them to change their eating habits (KK6).	2.54
	When I go for shopping with my mother or father, I buy healthy snacks such as nuts, raisins and dried chickpeas instead of chips, snacks or chocolate (KK7).	2.14
	I manage my schedule in the way to be able to do exercise for half an hour every day (KK8).	2.25
**Dependent variable**		
Nutrition knowledge§	Use salt or sodium only in moderation (NKB1)	3.08
	Have breakfast as it is the most important meal of the day (NKB2)	3.57
	Choose a diet with plenty of fruits and vegetables (NKB3)	3.46
	Use sugars only in moderation (NKB4)	3.03
	Choose a diet with adequate fibre (NKB5)	3.08
	Eat a variety of foods (NKB6)	3.11
	Maintain a healthy weight (NKB7)	3.51
	Choose a diet low in fat (NKB8)	3.38
	Drink at least six glasses of water (250 mL/glass) each day (NKB9)	3.53
	Choose a diet with plenty of breads, cereals, rice and pasta (NKB10)	3.26
	Eating meat is important because it is richer in protein (NKB11)	3.42
	Fruit and vegetable consumption as part of a heaIthy diet (NKB12)	3.33

† , NLU1 & NLU2 measured as 1= yes and 2 = no; NLU4 measured as 5 = always, 4 = often, 3 = sometimes, 2 = never and 1 = nutrition label do not influence my choice; NLU5 to NLU7 measured as 4 = very important, 3 = somewhat important, 2 = not too important and 1 = not at all important; NLU8 to NLU10 measured as 5 = strongly agree, 4 = agree, 3 = neutral, 2 = disagree and 1 = strongly disagree.

‡ , Know_a1 to Know_a11 measured as No = 1 and Yes = 2; KK1 to KK8 measured as 3 = always, 2 = sometimes and 1 = not at all.

§ , NKB1 to NKB12 measured as very important = 4, somewhat important = 3, not too important = 2 and not at all important = 1.

Table 2 lists the responses regarding the nutrition label use scale. Results in Table 2 concerning the question of food label awareness reveal that almost 61% of the youth are unaware of the contents on food labels. This could explain why 67% of the youth indicated that they read the nutrition information on the labels before placing food products into their shopping baskets. The 6% difference could be a result of using a dichotomous scale where ‘yes/no’ options were given. This accommodates those who may not be sure if they are fully aware of food labels and hence the 6% discrepancy. The thinking here is that the youth are eager to know the nutritional value of the food items before they consume. When asked how often they choose products with nutrition labels over products without labels, the majority of the respondents indicated that sometimes they do (42%), with 22% indicating never and 23.3% indicating that nutrition labels do not influence their choices. Only 9% of the respondents indicated that they choose products with nutrition labels over products without labels. When asked how important nutrition information about food ingredients is when doing shopping, 64% of the youth stated that it is very important and only 8.7% said it is not too important.

**TABLE 2:** Distribution of responses (%) to the label use questions.

Responses	Question								
	NLU1	NLU2	NLU4	NLU5	NLU6	NLU7	NLU8	NLU9	NLU10
Yes	39.3	67.3	-	-	-	-	-	-	-
No	60.7	32.7	-	-	-	-	-	-	-
Always	-	-	9.3	-	-	-	-	-	-
Often	-	-	3.3	-	-	-	-	-	-
Sometimes	-	-	42	-	-	-	-	-	-
Never	-	-	22	-	-	-	-	-	-
Nutrition labels do not influence my choice	-	-	23.3	-	-	-	-	-	-
Very important	-	-	-	64	54.7	91.3	-	-	-
Somewhat important	-	-	-	25.3	24	8.7	-	-	-
Not too important	-	-	-	8.7	21.3	-	-	-	-
Strongly agree	-	-	-	-	-	-	62	83.3	72.7
Agree	-	-	-	-	-	-	23.3	16.7	18
Neither agree nor disagree	-	-	-	-	-	-	11.3	-	7.3
Disagree	-	-	-	-	-	-	2	-	2

NLU, Nutrition label use understanding.

The results in Table 2 also indicate that 54.7% of the respondents consider standardised labelling on food packages as very important, with 21.3% of the respondents considering it as not too important. In addition, 91.3% of the respondents indicated that when doing their shopping, production and expiration dates of commodities are very important items and 8.7% indicated that they are somewhat important. It is further deduced from Table 2 that amongst the youth, nutrition information plays a critical role in determining the food items they buy (62% of them strongly agreed, 23.3% agreed, 11.3% neither agreed nor disagreed and only 2% disagreed). In addition, it is evident that the majority of the youth consider reading the production and expiration dates on food packages as important for an individual’s health, as represented by 83.3% of respondents who strongly agreed and an additional 16.7% who agreed. Finally, 72.7% of the youth also strongly agreed that unhealthy food packaging without a standard sign and health licence should not be used, 18% of the youth agreed, and only 7.3% neither agreed nor disagreed.

As mentioned earlier, the reading of the nutrition information scale was made up of two parts, with the first part comprising 11 questions measured on a dichotomous scale. From this information, a total score for each respondent could be computed. The minimum score would be (1 × 11 = 11) and the maximum score would be (2 × 11= 22). From the observed scores in Table 1, the total score for the youth is 18.68, with a mean score of 1.7. Although the value of 1.7 is close to 2 (on the mentioned dichotomous scale), the information indicates that the youth’s score for reading nutrition information is above average. The second part of reading nutrition information had eight items measured on a three-point Likert scale. A total score was computed for this scale, with the minimum possible score established as 8 (8 × 1= 8), the maximum possible score established as 24 (8 × 3= 24) and the observed score as 16.8, with a mean score of 2.1. From the observed scores, it can be concluded that the youth scored far above the average score for reading of nutrition information.

Finally, the total score for the nutrition knowledge scale was computed with the goal of determining whether the youth had a high or low nutrition knowledge score. A similar approach in computing the total score was used. Thus, the minimum possible score of 12 (12 × 1=12) could be obtained given that the nutrition knowledge scale had 12 items measured on a four-point Likert scale, with 4 = very important and 1 = not at all important. The maximum possible score would be 48 (4 × 12 = 48). Table 1 shows that a total of 39.76, far above average, with a mean of 3.31 was scored. These results also indicate that the youth had a high score for nutrition knowledge. Barreirro-Hurle et al. (2010) validated the measures mentioned above through pilot surveys.

The study’s first hypothesis sought to investigate if there is a significant difference between men and women regarding nutrition knowledge. Table 3 reports on the results of this analysis. The independent samples *t*-test was undertaken and the mean score for men was 3.35, with a standard deviation (SD) of 0.28. The mean score for women was observed as 3.30, with an SD of 0.31. Equal variances were assumed, with *F* = 1.223, Sig. (*p*) = 0.271 giving the *t*-value = 1.024 and degrees of freedom = 141.000. From these figures, a *p*-value of 0.308 was obtained, indicating that the difference between means was not significant. In other words, amongst the youth in this study, the nutrition knowledge of men and women does not differ significantly.

**TABLE 3:** Independent samples *t*-tests descriptive statistics and significance test output for gender.

Nutrition knowledge	Statistics	Sample size	Mean	SD	Std. error estimation
**Gender**					
Male	-	62	3.3454	0.28304	0.3595
Female	-	81	3.2942	0.30593	0.3399
Equal variances (*F*)	1.223	-	-	-	-
Sig (*p*)	0.271	-	-	-	-
*T*-value	1.024	-	-	-	-
*df*	141.01	-	-	-	-
*p*-value	0.308	-	-	-	-

SD, standard deviation; Std. error, standard error.

The second hypothesis sought to determine whether the number of years of tertiary education has a significant effect on the nutrition knowledge of the youth. The thinking here is that as the youth complete more years at a tertiary institution, their nutrition knowledge should increase proportionately. In other words, those who have only completed a few years of tertiary education will most likely have less nutrition knowledge than those who have studied for longer. To investigate this hypothesis, a one-way independent ANOVA was undertaken. The ANOVA output (descriptive section) shows that first year students had a mean score of 3.43, SD of 0.193 and a standard error (SE) of 0.064. The second year students had a mean score of 3.230, SD of 0.31 and SE of 0.07. Finally, the third year students had a mean score of 3.330, SD of 0.260 and SE of 0.04. To determine if these differences between the four groups were significant, the following figures were observed: *F* (3.135) = 1.347, *p* = 0.262. This is reported in Table 4a and Table 4b. (Based on the responses we received on the question regarding education level, the results indicated that *N* = 139, meaning that out of the original *N* = 150 students, 11 did not provide meaningful data on this question.)

**TABLE 4a:** Analysis of variance descriptive statistics and significance test output for years of study.

Year of Study	Sample Size	Std. Deviation	Std. Error
1st Year	9	0.19295	0.06432
2nd year	19	0.31004	0.07113
3rd year	59	0.25622	0.03336
4th year	52	0.26453	0.03668
**Total**	**139**	**0.26493**	**0.02247**

Based on the responses we received on the question regarding education level, the results indicated that (*N* = 139, meaning that out of the original *N* = 150 students, 11 did not provide meaningful data on this question).

**TABLE 4b:** Analysis of variance descriptive statistics and significance test output for years of study.

Statistics	Significance
Levence statistic	1.482
df1	3
df2	135
sig	0.222
Sum of Squares between Groups	0.282
Sum of Squares within Groups	9.405
*F*	1.347
Sig	0.262

Based on the responses we received on the question regarding education level, the results indicated that (*N* = 139, meaning that out of the original *N* = 150 students, 11 did not provide meaningful data on this question).

The descriptive statistics and common knowledge point to the notion that as someone completes more years of education, the likelihood of having more nutrition knowledge is greater for that person. However, the findings of the above ANOVA test indicated otherwise and there could be various reasons for this. One important possibility could be that the study respondents relied more on nutrition knowledge they had obtained during the adolescence stage and their tertiary education was not at all related to nutrition. Another reason could be that the respondents were majoring in disciplines not related to the health sciences. This could be that the campus in which the students were based did not offer health sciences-related courses.

To investigate whether nutrition label use and reading of nutrition information predict nutrition knowledge, a multiple linear regression was undertaken (see Tables 6–8). A positive but non-significant Pearson’s correlation was observed between the predictor variables and the explanatory variable. Thus, a correlation of 0.088 was observed between nutrition label use and nutrition knowledge. This was observed through the regression analysis (*r* = 0.092) between reading of nutrition information and nutrition knowledge.

**TABLE 5:** Multiple linear regression output: Pearson’s correlation results regarding nutrition knowledge and label reading.

Correlations	Nutrition_Knowl_Belief	Nutrition_LU5_7	Reading_of_NInfor
**Pearson correlation**			
Nutrition_Knowl_Belief	1.000	0.088	0.092
Nutrition_LU5_7	0.088	1.000	0.154
Reading_of_NInfor	0.092	0.154	1.000
**Sig. (1-tailed)**			
Nutrition_Knowl_Belief	-	0.159	0.148
Nutrition_LU5_7	0.159	-	0.040
Reading_of_NInfor	0.148	0.040	-
***N***			
Nutrition_Knowl_Belief	131	131	131
Nutrition_LU5_7	131	131	131
Reading_of_NInfor	131	131	131

**TABLE 6:** Multiple linear regression output – Model summary results.

Model	*R*	*R*-square	Adjusted *R*-square	Std. error of the estimate	*R*-square change	Change statistics				Durbin–Watson
						*F* change	df1	df2	Sig. *F* change	
1	0.118†	0.014	−0.001	0.25426	0.014	0.909	2	128	0.406	1.890

Note: Model Summary, Dependent Variable: Nutrition_Knowl_Belief.

† , Predictors: (Constant), Reading_of_NInfor, Nutrition_LU5_7.

**TABLE 7:** Multiple linear regression output – Analysis of variance results.

Model 1	Sum of squares	df	Mean square	*F*	Sig.
Regression	0.118	2	0.059	0.909	0.406†
Residual	8.275	128	0.065	-	-
**Total**	**8.392**	**130**	**-**	**-**	**-**

Note: ANOVA, Dependent Variable: Nutritionon_Knowl_BeIief.

† , Predictors: (Constant), Reading_of_NInfor, Nutrition_LU5_7.

**TABLE 8:** Multiple linear regression results – Coefficients.

Model 1	Unstandardised coefficients		Standardised coefficients Beta	*t*	Sig.	95.0% confidence interval for B		Correlations			Collinearity statistics	
	*B*	Std. error				Lower bound	Upper bound	Zero-order	Partial	Part	Tolerance	VIF
(Constant)	2.991	248	-	12.050	0.000	2.500	3.483	-	-	-	-	-
Nutrition_LU5_7	0.42	0.49	0.076	0.850	0.397	−0.056	0.140	0.088	0.075	0.075	0.976	1.024
Reading_of_NInfor	0.87	0.96	0.080	9.03	0.368	−0.103	0.276	0.92	0.080	079	0.976	1.024

Std. error, standard error; VIF,.

Note: Coefficients, Dependent variable: Nutrition_Knowl_Belief.

To determine if the explanatory variables had a unique contribution towards the model, results from the model summary table were examined and an *R*-square = 0.0140 or 1.4% of the variance in nutrition knowledge is explained by reading of nutrition information and nutrition label use. Based on the data analysis, it can be observed that nutrition label use and reading of nutrition information do not predict nutrition knowledge as found amongst the participating respondents of the study.

Furthermore, the ANOVA results (from the multiple linear regression output) showed the results which explain whether the use of the model was significantly better at predicting nutrition knowledge of the youth. Given F (0.909) with a significance of 0.406, the model was declared unfit. In other words, the model has no value in predicting youth nutrition knowledge. The *B*-values showed that a positive but non-significant relationship exists between nutrition label use and nutrition knowledge (*B* = 0.042; *p* = 0.397). Also, a positive but non-significant relationship was confirmed between reading nutrition information and nutrition knowledge (*B* = 0.087; *p* = 0.368).

## Discussion

The aim of this study was to measure the relationship between nutrition label use and nutrition knowledge. Three main findings of this study are reported in this section. Firstly, rural youth consumers’ nutrition label use had no significant relationship with nutrition knowledge. Secondly, no significant difference existed by gender concerning nutrition knowledge. Finally, the level of education amongst the rural youth consumers studying at a university had no relationship with aspects of nutrition knowledge.

Generally, the study supports the notion that contextual issues appear to influence behaviours concerning nutrition knowledge (Kabir et al. 2018). Uniquely, the study illustrates this within a South African rural context. Furthermore, the study shows support of previous work that investigated the influence of demographic variables on nutrition label use (Psaltopoulou et al. 2017). It is encouraging, based on the finding, to note that gender has no influence on the relationship between nutrition label use and nutrition knowledge, albeit in South Africa where gender inequality exists. Of concern, however, is the finding that the level of education (measured by the number of years of tertiary education) did not relate to aspects of nutrition knowledge. Despite strides by countries, including South Africa, in publishing food-labelling legislation (Kempen et al. 2012), there appear to be challenges with regard to the influence of food labels on nutrition knowledge. Whatever the amount of educational progress, nutrition labels do not appear to be prioritised. This could be a familiarity that exists regarding this activity. Therefore, the issue is not so much about encouraging legislation that protects the consumer, as is the case globally (Ishak & Zabil 2012), but changing attitudes of the intended beneficiary of such edicts, the consumer. Hence, efforts to change attitudes may improve decisions about reading nutrition labels (Miller et al. 2017) and inform on aspects of nutrition knowledge (Miller & Cassady 2015).

## Recommendations

Apart from advancing theory, marketers can benefit from the findings of this study. Marketers need to consider the factors that influence nutrition label use and nutrition knowledge amongst the youth as active consumers. This then can be a basis for encouraging interventions that not only promote responsible product use but also the health of the consumer. Based on the findings of the research, programmes can be encouraged to assist communities in better understanding the information on labels of products, given the link between nutrition label use and nutrition knowledge. It is envisaged that such a study and its implications can be a major means of encouraging consumers in making healthier choices.

## Limitations and future research

The results of this study cannot be generalised to the entire population of youths that reside in rural communities or any other context. However, the results serve as a useful guideline for understanding issues concerning nutrition label use and nutrition knowledge. It is envisaged that this can be a useful precursor for interventions in improving sustainable livelihoods. Future research expanding on the findings of this quantitative study should be undertaken. For instance, such research could seek to explore further determinants of nutrition label use amongst youths against (1) the type of product, (2) frequency of use, (3) the role of the sociocultural milieu and even (4) culture. Future research should also take a qualitative stance in exploring the complexity that may accompany nutrition label use and reading and nutrition knowledge. Finally, consideration should be given to collecting data at multiple points, thus giving more robust data for understanding issues around nutrition label use and nutrition knowledge.
